# Circumstances and Consequences of Snakebite Envenomings: A Qualitative Study in South-Eastern Costa Rica

**DOI:** 10.3390/toxins12010045

**Published:** 2020-01-11

**Authors:** Jazmín Arias-Rodríguez, José María Gutiérrez

**Affiliations:** Instituto Clodomiro Picado, Facultad de Microbiología, Universidad de Costa Rica, San José 11501, Costa Rica

**Keywords:** snakebite envenomings, poverty, amputations, antivenom, public health, rehabilitation

## Abstract

A qualitative study was carried out in south-eastern Costa Rica on the circumstances and consequences of snakebite envenomings. This region has the highest incidence of snakebites and the lowest per capita and per family income in the country. There is a high degree of destitution and an unstable labor situation in the region. This study was based on semistructured interviews with 15 people who had suffered snakebite envenomings. This sample size was established on the basis of data saturation. Bites occurred mostly while doing agricultural work, either as salaried workers, as occasional workers, or working on their own. Although all people were attended in health centers of the public health system, and received antivenom free of charge, the majority of them did not receive compensation or rehabilitation upon discharge from the health facilities as a result of not being regular salaried workers. People described many difficulties as a consequence of these envenomings, such as permanent physical sequelae, including two amputations, psychological consequences, economic hardships, and difficulties for reinsertion into agricultural work. In spite of the significant advances that Costa Rica has made for reducing the impact of these envenomings, results reveal issues that require urgent attention by government and civil society organizations, to compensate for the physical, psychological, social, and economic consequences of these envenomings.

## 1. Introduction

Snakebite envenoming is a neglected tropical disease that exerts a high burden of morbidity and mortality, particularly in sub-Saharan Africa, Asia, Latin America, and parts of Oceania [1,2]. It is estimated that every year, there are 1.8 to 2.7 million envenomings worldwide, with an estimated number of 81,000 to 138,000 fatalities, and ~400,000 people left with permanent physical and psychological sequelae [2]. Snakebites affect predominantly persons living in impoverished rural communities [3,4]. Owing to these envenomings affecting the ability of people to perform agricultural duties and other activities, as well as to the costs involved in the treatment of the cases, snakebites cause a vicious cycle of poverty, generating adverse social impacts. The heavy socioeconomic consequences of envenomings for patients and their families have been highlighted in several studies [5,6]. 

One aspect of this health problem that has received relatively little attention from a research perspective is related to the various consequences generated by these envenomings. Owing to the tissue-destruction effects of many snake venoms, particularly those of species of the family Viperidae and some species of the family Elapidae, as well as to the deleterious actions of these venoms in a number of organs, a percentage of envenomed people are left with permanent physical sequelae of various types, such as tissue loss, amputations, contractures, arthrodesis, septic arthritis, hypertrophic and keloid scars, tendon damage, blindness, chronic skin ulcers, osteomyielitis, chronic renal failure, and neurological sequelae, among others [2,7,8,9,10,11,12,13]. In addition, a high incidence of psychological sequelae has been described in various studies [14,15]. The physical and psychological effects of envenomings may lead to social stigmatization and may have a profound effect on the quality of life of affected people and their families. 

Snakebite envenomings occur in particular socioeconomic and cultural contexts, which interact, in a dynamic and complex fashion, to generate conditions for the bites to occur, and which affect the outcome of the envenoming in terms of management of the case and its long-term consequences. It is therefore of relevance to study the circumstances and consequences of snakebite envenomings in various contexts, as stressed in the recently launched World Health Organization (WHO) strategy for the prevention and control of these envenomings [16]. To this end, the incorporation of social research in the study of this neglected tropical disease could provide renewed light for understanding its highly complex landscape, which goes well beyond the epidemiological and biomedical aspects [17]. 

Health issues can be studied from an ‘external’ perspective, i.e., by describing the epidemiological and clinical features of diseases. In addition, illnesses can be analyzed from an ‘internal’ perspective, i.e., based on the self-perception of the patients. This latter perspective better captures the centrality of suffering in the disease process and its consequences [18]. Qualitative research based on in-depth interviews is a valuable analytical tool to investigate snakebite envenomings from this ‘internal’ perspective, as it provides clues for a more holistic analysis of the multiple angles associated with this disease. It is worthy to note that there is a scarcity of qualitative studies focused on snakebite envenoming.

In Costa Rica, between 400 and 600 snakebite cases are reported every year [19]. The average incidence for the period 1990–2007 was 13.8 cases per 100,000 people per year [20]. The highest incidence of snakebites occurs in the province of Puntarenas, particularly in the south-eastern region of this province, where incidences higher than 100 cases per 100,000 people per year have been described in some localities [20]. In the socioeconomic division of this country, this region is known as the Brunca Region, which encompasses five ‘cantones’ (local political units) in the province of Puntarenas and one in the province of San José [21]. In addition to having a high incidence of snakebites, this region has the lowest per capita and per family income, being the second poorest region in the country [22]. Hence, it combines a high incidence of snakebites with very low socioeconomic parameters, hence constituting a setting of high vulnerability for snakebite envenomings. 

A qualitative ethnographic study was developed in the Brunca Region of Costa Rica, based on semistructured interviews with people who had suffered snakebite envenomings. The study focused on the circumstances of the bites, on the physical, psychological, and socioeconomic impacts of these accidents on the affected people, and on the attention received from the public health system of the country. 

## 2. Results and Discussion

### 2.1. General Characteristics of the People Interviewed

Fifteen people were interviewed for this study. Interviews included personal features, labor conditions, circumstances of the snakebite, and main consequences of the envenomings. They were 13 men and two women, whose ages at the time of the bite ranged from 12 to 69 years. The snakebites occurred between 1995 and 2015. At the time of the interviews ages ranged from 20 to 83 years. 

### 2.2. Identification of Nodes of Analysis

The analysis of the interviews, using the methodology described in the Materials and Methods Section, allowed the identification of the following main nodes of analysis: (a) Sociodemographic and economic conditions that determine the vulnerability of people to suffer a snakebite; (b) attention provided after the bite by the public health system; and (c) consequences of the snakebite envenoming. Before the analysis of the interviews, a brief historical description is presented on the economic dynamics of the Brunca region in order to contextualize the setting where the study was carried out.

### 2.3. Economic Dynamics in the Brunca Region of Costa Rica

Before the decade of 1930, the Brunca region of Costa Rica had been populated by various indigenous ethnic groups, and by migrant peasants from the central part of the country and from the region of Chiriquí in neighboring Panama; this region developed a predominantly subsistence type of agriculture [23]. In the 1930s, a radical change in the use of land occurred in the region, with the development of extensive banana plantations by the multinational United Fruit Company (UFCo, New Orleans, Louisiana, USA). Since then, the evolution of the economic dynamics of large parts of the region has been characterized by a steady process of land concentration by agroindustrial complexes [23]. This represented a radical shift in land use and property, since the UFCo acquired a high proportion of the land in the central Pacific and the Brunca regions of Costa Rica. In this process, small rural properties were displaced, and many people who used to own their land became dispossessed, many of whom underwent a process of proletarianization and impoverishment [23]. This process, which occurred in many countries in Latin America and elsewhere, has been dubbed ‘accumulation by dispossession’ [24]. In the decade of 1980, for diverse reasons, the UFCo largely abandoned its economic activities associated with banana cropping in the south-eastern region of Costa Rica, generating an economic crisis associated with a high unemployment rate [23].

An economic reconversion occurred in the region with a transition from extensive banana plantation to large-scale cultivation of oil palm. This had been initiated on a small scale in the previous decades, but was then incremented by the UFCo/United Brands, and was greatly expanded in the decade of 1980 and afterwards, with a dominant role of another large company, Palma Tica (Puntarenas, Costa Rica) [25]. In addition, in the last decades, local cooperatives, such as Coopeagropal and CIPA (Puntarenas, Costa Rica), and small scale producers have also been involved in the production and industrialization of oil palm in this region [25]. However, the monopolistic pattern that began with bananas decades ago was largely maintained with this new predominant crop in the region [25]. Noteworthy, several salaried workers interviewed for this study were employees of this oil palm company. 

From an ecological perspective, the expansion of banana and oil palm plantations caused a variety of environmental alterations. As one of the consequences of this phenomenon, venomous snakes and other wildlife species, such as the ‘terciopelo’ (*Bothrops asper*), which has a great ecological versatility and is the most important snake in the country from the medical point of view [26], invaded and adapted to these plantations and to nearby human settlements, with the consequent risk for rural workers.

### 2.4. Sociodemographic and Economic Conditions That Determine the Vulnerability of People to Suffer a Snakebite

Of the 15 persons interviewed, 11 were doing agricultural work at the time of the bite. Among them, five were permanent salaried workers, four were workers contracted informally, and two were working by their own in their small properties. The rest (four people) were not doing agricultural work strictly speaking, but were carrying out other duties nearby their houses or in a biological station, such as cutting grass, cleaning the land, or in one case, doing housework. The high proportion of people performing contracted agricultural work, either under formal or informal labor conditions, is in agreement with a general trend in the rural areas of Costa Rica and elsewhere in Latin America, whereby a majority of people do not own their own land and rely, to a large extent, on unstable jobs associated with a low income [27]. The observation of one bite occurring inside a home points to the feasibility of domiciliary accidents, particularly in the case of *B. asper*, which adapts to live nearby human dwellings [26]. In addition, the high prevalence of destitute housing in this impoverished region facilitates the entrance of snakes to homes, enhancing the risk of a bite [4]. Despite the fact that previous epidemiological studies indicate that the majority of snake bitten people in Costa Rica are young agricultural workers [19,28], six participants were more than 60 years old when bitten, probably reflecting a demographic trend in rural areas of Costa Rica towards an increasing proportion of older people [29]. Only two out of 15 participants were women, in agreement with the general trend in Costa Rica of higher incidence of snakebites in men [19,28].

Six out of 11 people who were involved in agricultural duties at the time of the bite were working in oil palm plantations, while the rest were working on beans, plantain, corn, or coffee crops. This trend is congruent with the predominance of oil palm plantations in the south-eastern region of Costa Rica. In 2014, there were 66,419 Ha of land in Costa Rica devoted to this crop, 87% of which were in the province of Puntarenas, where our study was carried out [29]. Oil palm plantations in Costa Rica pose a risk for snakebites owing to the frequent presence of *B. asper* in these areas (our unpublished observations, Instituto Clodomiro Picado, San José, Costa Rica). The snakebites occurred between 06:00 and 15:00, corresponding to the time of the day when agricultural labor is done. Most affected people referred to being the only source of income in their families, hence reinforcing the economic and social impact of these accidents. 

Seven of the affected people were working alone at the time of the bite. They emphasized the need to work with other persons, so that they could rapidly communicate the occurrence of the accident, facilitating the rapid transportation of the victim. Eleven interviewees were wearing boots, usually rubber boots, at the time of the bite, and in these cases bites occurred in the upper segments of the lower extremity or in the hands. This finding stresses the fact that, although about half of snakebites in Costa Rica occur in the feet [28], snakes, especially large ones like *B. asper*, are able to bite in other anatomical regions, mostly the hands. 

A consistent finding was the perception that the companies or landowners for whom these people were working do not provide enough attention to the risk of snakebites. Little information for preventing these accidents is offered, and few educational campaigns on snakebites are organized by these employers. In such contexts, people often receive incorrect information about this topic, thus generating confusion, as two of the participants declared:

 “The men lifted me and brought me to an old house, and applied a tourniquet…and it hurt a lot, and I wished they would take out the tourniquet, and then they took me to the hospital.”

(TA-ENT06)

 “Yes, information on how to act is needed. The only thing I knew was from a person who told me ‘when you get bitten by a snake, take it, get out its liver and bile and eat them, and you will see that nothing happens to you’. The company does not provide information.”

(TA-ENT04)

This contrasts with efforts carried out by Instituto Clodomiro Picado (Universidad de Costa Rica, San José, Costa Rica) through its extension programs in rural communities, and by other stakeholders in the country, which have contributed to the increased awareness on the risk of snakebites and on the appropriate types of actions to be taken after a bite, as reflected by the low percentage of affected people in the country who apply nonrecommended first aid measures [28]. Our observations in this study, however, highlight the need to further strengthen these campaigns, with the necessary involvement of the private sector and employers in rural settings.

Regarding the perception concerning the feasibility of preventing snakebites, participants considered that it is not possible to guarantee a complete prevention of these accidents, even when measures, such as using boots and being careful while doing agricultural work, are implemented. Some of the people interviewed were bitten by specimens of *B. asper* above the knee, despite wearing boots. The following statement illustrates this point:

 “Well, in the field the only prevention is to wear boots, but in my case the snake bit me above the boot, there was little else to do because if you wear boots and the animal is large it can bite above the boots and there is no way to avoid it.”

(TA-ENT03)

### 2.5. Attention Provided after the Bite and by the Public Health System, and Sequelae of Envenomings

Most people interviewed complained about the poor attention after the accident by the company or the landowners for whom they were working. This was more evident in the cases where the affected person was hired as an occasional worker, as evidenced in the following statement:

 “The man for whom I was working did not even give me a glass of water during the two months I could not work, he did not even ask whether I had died or not.”

(TA-ENT03)

Social health insurance is mandatory for all workers, whether salaried or self-employed. Dependent family members and people in need are entitled to health insurance as well. The medical attention provided by the public health system (Caja Costarricense del Seguro Social-Costa Rican Social Security System (CCSS)) does not depend on the type of access to social insurance, contributory or noncontributory, salaried or nonsalaried, of the person at the time of the bite. Occupational hazards insurance, taken care of by Instituto National de Seguros (National Insurance Institute, (INS)), instead, is mandatory for salaried workers only. 

In the cases of the people interviewed for this study, all of them received the medical attention free of charge at the CCSS, regardless of their particular legal labor status. Among the four people who were not doing agricultural work when bitten, all of them were covered by the CCSS; one was directly affiliated to the CCSS and three benefited from a family affiliation system.

On the other hand, only permanent workers on a payroll are entitled to the benefits of the Law of Labor Risks of the INS, i.e., specialized medical attention, rehabilitation, and compensation. This creates a deep differentiation in the attention of people suffering snakebite envenomings depending on the type of labor arrangements. People hired informally as occasional workers are generally not benefited by the Law of Labor Risks of INS, despite comprising a large group of salaried agricultural workers in this region of Costa Rica; this has a direct impact in the long-term consequences of this type of labor accident. Among the 11 people bitten while doing agricultural work, six did not get the INS labor policy for not being formal salaried workers. This situation needs to be considered by health authorities and policy makers in Costa Rica, since the current regulations leave a large number of rural people working in unstable labor conditions or on their own, in a highly vulnerable situation in terms of a lack of public support when facing the consequences of envenomings beyond medical attention. Changes in the legislation are necessary in order to include income replacement and rehabilitation to these people.

The majority of interviewees were rapidly transported to the nearest health facility (hospital, clinic, or primary health post), and all of them received treatment, including antivenom, at the CCSS. Three of them arrived in less than 30 min, five in less than 1 hour, six in 2 hours, and one took 6 h to reach a health facility. In most cases, the health center was located less than 10 km from the site of the accident, although in three cases the center was 30 to 50 km away. Two persons were transported in ambulances, whereas the rest reached the health posts either by walking, or by bus, car, or boat. Eleven persons considered the hospital attention they received to be satisfactory, whereas four of them complained about the delay in the attention upon arrival, and on the fact that, for a number of reasons, they had to be transported to another health center. Owing to the complications of the bite, one person was taken to four different hospitals, ending in Hospital San Juan de Dios in the capital city of San José. Despite the observation of the relatively close location of health facilities in the region, some people complained about the difficulties of reaching such facilities, or the fact that in some health posts, attention is not provided 24 h a day, as expressed by one patient:

 “…The closest locality here is Puerto Jiménez, because in my community there is a clinic, but it is not open 24 h, it opens in working hours only; if you go now, it is probably closed, there is nobody there. Thus, the closest place is Puerto Jiménez…If it rains that is another thing, the rivers overflow, and the situation gets more complicated, it is 80 km away.”

(NTA-ENT03)

A previous study identified several localities in the south-eastern region of Costa Rica as being more than 3 hours away from the nearest health center [20]. This calls for a detailed analysis of aspects such as schedules of attention and effective transportation systems to ensure that people suffering a snakebite, or other types of emergencies, can be rapidly transported to health posts and receive rapid and effective interventions.

### 2.6. Consequences of the Snakebite Envenoming

#### 2.6.1. Physical and Psychological Sequelae

Participants expressed that the snakebite caused physical consequences and sequelae of various types. Ten people described their snakebite envenoming as severe, and the same number perceived that the accident greatly affected their life in several aspects. Eight people described having difficulties moving around after leaving the hospital and in the following months or years. Twelve persons declared that the consequences of envenoming jeopardized their labor capacity and skills afterwards. Two suffered amputations (leg and arm), which brought many consequences in their everyday life. The following testimony illustrates these findings:

 “Sure, it makes sense. It is something that you cannot get rid of easily, it will remain your whole life, because losing a leg, for example… And also you have to take care, do not walk too long because although the venom is gone, some deficiencies remain, deficiencies that affect you, you know?”

(TA-ENT05)

In addition, nine people described having psychological consequences. The following outcomes were described: (1) Recurrent dreams with snakes; (2) intensified fear of snakes, or emergence of fear of snakes; (3) depression, anxiety, and irritability; (4) intense fear of suffering another snakebite; and (5) other mental problems. These types of sequelae are clearly expressed in the following statements:

 “It was very tough, I became so stressed that I almost died, I was in very bad shape…and on top of that my partner was in jail, I said how terrible, I am worthless… and I said God bless, I am worthless, I cannot do anything…it was terrible.”

(NTA-ENT01)

 “Well, the hiccups did not come back, but often one feels discouraged, it’s the venom, like now I feel like lazy, like tired, like I need to rest…The snake bitten person will always have problems, like in my case it was the hiccups, but other people have other things. In my case, when the snake bit me, it felt like an electric shock.”

(TA-ENT05)

The descriptions of these psychological consequences agree with previous studies in Sri Lanka [14,30] and Nigeria [15], which reported a variety of psychological outcomes secondary to snakebites, including anxiety, depression, and post-traumatic stress disorder. This issue deserves further investigations since it has been largely overlooked, not only in terms of research, but also in the lack of attention by public health systems. The WHO strategy for prevention and control of snakebites underscores the need to provide effective follow-up for people affected by these envenomings, not only in terms of providing rehabilitation and support for physical disabilities, but also by considering psychological support [16]. It is necessary to implement interventions of various sorts to ensure that people who have suffered a snakebite envenoming receive the appropriate follow-ups after their discharge from health facilities. This should involve not only the public health institutions, but also various types of civil organizations [31].

#### 2.6.2. Long-Term Socioeconomic Consequences

The participants, especially those who were doing agricultural work at the time of the bite, underscored the immediate economic impact suffered as a consequence of the accident. Since most of them were the only source of income for their families, this affected their whole family. This stresses that snakebite envenomings cause an expanding wave of social suffering that hits not only the bitten person, but also their families, and beyond that, their communities [32]. These people belong to a highly vulnerable social group, particularly those working in unstable labor conditions, and the snakebite further deepened their vulnerability and poverty. In some cases, the affected persons had to sell animals and belongings in order to cope with the economic needs. The following statements support this assertion:

 “Almost 2 years. At that time I had cattle, I was economically more or less stable and had pigs. I had pigs, cattle, dairy cattle, but as a consequence of the snakebite I had to start selling, selling, selling, and now I have nothing, I lost everything. Neighbors, friends, relatives helped me many times with food and economically because I was in bad shape, I was in a very critical situation, and I got help from many people…”

(TA-ENT05)

 “Well…my family suffered because they had to support me economically. I could not work, I could not provide anything to my people.”

(TA-ENT03)

This agrees with studies carried out in several Asian countries, where the cost of treatment forced people to acquire loans and sell family assets [5,6,33]. In the case of Costa Rica, the social public health system (CCSS) provides antivenom and medical care free of charge to most of the people affected, but nevertheless, there are other consequences leading to expenses that have to be taken care of by the patients and their families. In addition, the limitations of affected people to continue working have direct economic consequences for their families.

In the case of women, and depending on their family situation, snakebite envenomings may force them into a spiral of poverty and dependence, especially when they are the only economic support of their families. This is the case of a woman who was left with a permanent physical disability in her hand that did not allow her to perform physical work and earn an income. In order to support her children, she decided to live with an older man, even though she was the victim of his psychological abuse.

 “…So, I had to come here, with this man, live with him because I did not have where to go with my children, they were in school and I did not have anywhere to go, and I said oh God I do not want them to quit school, because you know how hard it is without schooling, I do not want them to quit, oh God, I said, how do I do it?”

(TA-ENT07)

The difficulties people had regarding a productive reinsertion into work were stressed in the interviews. Of the 15 people, only one returned to work within 3 days, because the envenoming was mild. Six people returned to work 2 to 3 months after the bite, and it took 5 to 6 months for five of them. This period lasted 1 year or more for two participants, and 3.5 years for another person. The time lapse between the bite and the return to work had economic and other implications, especially when the bitten person was the only economic support for the family. In some cases, the land owners for which the persons worked refused to rehire them because of their physical disabilities, as indicated by the following statement:

 “I was not able to work for six months, seven months…I went back to the farm where I worked when my incapacity license ended, I went back there and I was told that I could not do any type of work there, they said, because with that hand you cannot work. He said to me that they could not give me any type of job, and that I could not go back to work. They did not pay me anything, that was all he said, that they could not offer me a job because I could not work, they did not give me even a piece of paper, some money, nothing, nothing.”

(TA-ENT07)

One person interviewed underscored the difficulties getting a job as a consequence of the snakebite:

 “Economically yes, it is difficult, because it is not the same when I was well as I am now, because the people who used to look for me to do some jobs, they no longer look for me because of the limitations I have…”

(TA-ENT-05)

These findings highlight the difficulties for impoverished rural workers to return to work and to continue earning their income after a snakebite, either because they are left with physical disabilities that make such a return difficult or because of the uncertainty of the labor conditions in the region greatly reduce their possibilities. This is particularly problematic in vulnerable social contexts, such as in the south-eastern region of Costa Rica. In this regard, we found the same trends in the difficulties experienced by people regardless of the year when they suffered the snakebite.

In this highly adverse context, some of the interviews revealed the existence of social and family support, which provided protection in such difficult conditions. These local communal networks, together with other types of organizations, play a significant role in the resilience that communities develop for dealing with health issues and other situations when public services do not offer the necessary coverage [31]. It is necessary to do research on these social networks in various cultural contexts, and to strengthen the empowerment and engagement of communities to confront the consequences of snakebites [16].

## 3. Conclusions

This qualitative study revealed the difficult situation experienced by people who suffer snakebite envenomings in rural settings in south-eastern Costa Rica, a region with a high degree of poverty and destitution, reflected by poor housing, low salaries, and unstable labor conditions. In this context, snakebite envenomings represent a public health hazard that causes drastic physical, psychological, social, and economic consequences, as demonstrated in this work. The narratives of the people interviewed provide compelling evidence of the human suffering generated by envenomings.

Despite the fact that Costa Rica has made significant developments in the control of snakebite envenomings, as reflected in a well-developed public health system; the availability of antivenom in health centers at primary, secondary, and tertiary levels which are provided free of charge to patients; physicians and nurses with adequate training to manage these envenomings; relatively rapid access to health facilities; and prevention campaigns at the community level [34], our findings reveal a number of issues in need of improvement. Some of these are as follows: (a) Better organization of working conditions so that people are not alone while doing agricultural duties; (b) improvements in the dissemination of information on what to do in the event of a snakebite; (c) community-based activities aimed at promoting prevention of snakebites in aspects such as house building, cleaning the areas around houses, wearing boots, and identifying areas of high risk in plantations; (d) introducing, in the health system, follow-ups for people that return to their homes with sequelae after hospital treatment; and (d) the promotion of new legislation aimed at guaranteeing rehabilitation and compensation for all people suffering sequelae after envenomings. In this regard, Instituto Clodomiro Picado has started discussions with legislators and health authorities in order to prepare legislation to cover this need. The public health system must ensure that no one suffering from a snakebite is left without protection, and receives appropriate compensation, after medical treatment. Likewise, agroindustrial companies, cooperatives, and farms in which agricultural workers are exposed to the risk of snakebites should have a higher commitment to the protection of their workers, including the regular organization of prevention campaigns, provision of adequate equipment for the avoidance of envenomings, keeping the fields clean, and providing insurance for all workers. Thus, improvements should be promoted in both the public and private sectors as to reduce the risk of snakebites and to ensure the adequate care of people suffering these envenomings.

## 4. Materials and Methods

### 4.1. Type of Study

This work was based on a qualitative research aimed at exploring the social, labor, and health issues associated with the occurrence of snakebites in the region, as well as on the analysis of the circumstances of these accidents. The technique of data collection was the semistructured interview, i.e., a face-to-face conversation between the interviewer and the interviewee in which previously designed questions were presented. At the same time, on the basis of the answers provided, new questions emerged in a dynamic interchange. 

### 4.2. Universe of Study

This study was developed in the Brunca socioeconomic region of Costa Rica. This includes several ‘cantones’ (local political units) of the south-eastern region of this country. A historical analysis of the economic dynamics of the Brunca region was carried out before the interviews in order to characterize the economic landscape where the snakebites occurred. The persons interviewed for this study were mostly agricultural workers who had suffered a snakebite. The first individuals who were interviewed were selected on the basis of previous information from a clinical investigation. The sample grew during the course of the study on a basis of the principle of gradual selection, i.e., people previously identified as having suffered a snakebite provided information of other people in their communities who had been also affected by the same type of event, hence, a ‘snowball’ effect occurred as regards the amplification of the sample. The criterion of data saturation was used to define the size of the sample [35]. After interviewing 15 people, a saturation effect was observed, i.e., no new information appeared in the interviews, so the size of the sample was fixed at that number. 

Participants in the study were men and women who worked either in the fields or in facilities for the processing of crops. The size of the areas where these people work ranged from small plantations to industrialized, large-scale farms. According to the type of labor relationship, the interviewees belonged to the following categories: (a) Workers who earned a salary (salaried workers). These can be divided into permanent workers and occasional laborers who are hired for specific periods of time, and whose income depends on the amount of work done, and not on the hours devoted to work; (b) independent agricultural workers, who do not receive a salary and instead earn their income on the basis of their own work, usually in their small own properties; (c) people who, when bitten by a snake, were not doing direct agricultural work, but were carrying out another type of activity, like cleaning tasks. 

### 4.3. Interviews and Analysis of the Information

The study was approved by the Committee of Final Graduation Projects of the School of Social Sciences of Universidad Nacional, Costa Rica. All participants in the study signed a consent form after being informed on the details and scope of the project, and agreed to the data collected being included in a publication. Confidentiality was guaranteed, and anonymity of all participants was kept throughout the study and in the preparation of this manuscript. The general issues included in the interviews, which correspond to the predefined axes of analysis, were: (a) Sociodemographic and economic conditions that determine the vulnerability of people to suffer a snakebite; (b) circumstances associated with the occurrence of the snakebite; (c) physical and psychological sequelae developed as a consequence of the envenoming; (d) socioeconomic consequences of the envenoming; (e) management of the cases by the public health system, both at the hospital level and after discharge from health facilities. 

The interviews were recorded and transcribed verbatim. The analysis was carried out using the Nvivo 11 software for qualitative data analysis. The steps followed in the analysis were as follows: (a) An initial reading of the transcriptions, followed by a process of automatic coding, grouping the answers within the predefined axes of analysis; (b) a second reading of the interviews and second coding. The predefined axes were translated into nodes to which the coded information was associated. At the same time, new nodes emerged from this analysis; (c) a final analysis was done by interpreting and contrasting the information gathered vis-à-vis the theoretical perspective of the study.
